# Diosgenyl 2-amino-2-deoxy-β-D-galactopyranoside: synthesis, derivatives and antimicrobial activity

**DOI:** 10.3762/bjoc.13.227

**Published:** 2017-11-01

**Authors:** Henryk Myszka, Patrycja Sokołowska, Agnieszka Cieślińska, Andrzej Nowacki, Maciej Jaśkiewicz, Wojciech Kamysz, Beata Liberek

**Affiliations:** 1 Faculty of Chemistry, University of Gdańsk, Wita Stwosza 63, 80-308 Gdańsk, Poland; 2 Faculty of Pharmacy, Medical University of Gdańsk, Hallera 107, 80-416 Gdańsk, Poland

**Keywords:** antimicrobial activities, D-galactosamine, diosgenin, glycosylation, saponin, tetrachlorophthalimido derivatives

## Abstract

The synthesis of diosgenyl 2-amino-2-deoxy-β-D-galactopyranoside is presented for the first time. This synthetic saponin was transformed into its hydrochloride as well as *N*-acyl, 2-ureido, *N*-alkyl, and *N*,*N*-dialkyl derivatives. Antifungal and antibacterial studies show that some of the obtained compounds are active against Gram-positive bacteria and *Candida* type fungi.

## Introduction

Saponins are steroid or triterpenoid glycosides found in various plants [1]. They produce a soap-like foam when shaken in aqueous solutions, which makes them useful as detergents, foaming agents and emulsifiers. Saponins are known for their structural diversity and a wide range of biological properties [2]. Among others, they display different pharmacological activities, particularly antifungal [3–6] and antitumor [7–9].

The aglycone part of a saponin is termed sapogenin. Diosgenin, yamogenin, tigogenin, smilagenin and sarsapogenin are the most abundant sapogenins in nature [10]. They are linked via a glycosidic bond to a sugar unit, mainly D-glucose. Diosgenyl glycosides constitute a very important group among spirostanol saponins. Diosgenin has a double bond between the C-5 and C-6 atoms of the spirostanol skeleton and can be found in combination with different sugars in *Costus, Discorea, Paris, Solanum, Yucca*, and *Trillium* plants [11]. The plants containing diosgenyl saponins are used in folk medicine in many countries. Such herbal medicines exhibit anti-inflammatory, antibacterial, antifungal, antiviral, diuretic and expectorant activities [2,11]. Importantly, these also help fight cancer cells [12–14].

Although saponins, in which D-galactose is directly bound with sapogenin are rarely found in plants, there are some examples of such galactosides. For example, timosaponin, isolated from an *Anemarrhena asphodeloides* plant, is a natural spirostanol saponin, which has D-galactose bound with sarsapogenin [15]. *Tribulus* plants are known to contain tigogenin linked with D-galactose [16]. Moreover *Smilacina atropurpureea*, *Solanum indicum*, and the genus *Yucca* contain smilacinosides and funkiosides [17], indiosides [18], and elephanosides [19]. All these saponins are glycosides, where diosgenin is attached directly with D-galactose.

Besides their isolation from natural sources, the chemical syntheses of saponins have been intensively investigated to evaluate and improve their pharmacological activities [20–22]. For example, in search of new variants of steroidal glycosides, diosgenyl 2-amino-2-deoxy-β-D-glucopyranoside was synthesized [23]. Modifications of the amino group in this synthetic saponin led to analogs with promising antitumor, antifungal and antibacterial activities [24–31].

In this paper, for the first time, syntheses of diosgenyl 2-amino-2-deoxy-β-D-galactopyranoside, its hydrochloride as well as *N*-acyl, 2-ureido, *N*-alkyl, and *N*,*N*-dialkyl derivatives are presented. These new synthetic saponins were tested for their antifungal and antibacterial activities.

## Results and Discussion

### Chemistry

**Scheme 1:**
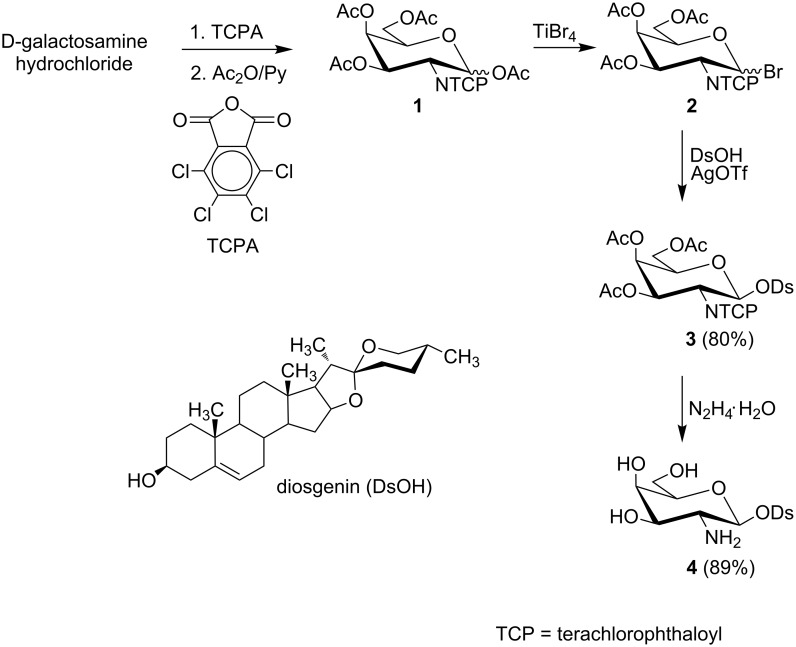
Synthesis of diosgenyl 2-amino-2-deoxy-β-D-galactopyranoside (**4**).

Relying on our previous experiences [30–31], we used the *O*-acetylated bromide **2** to synthesize diosgenyl 2-amino-2-deoxy-β-D-galactopyranoside (**4**) (Scheme 1). This glycosyl donor was *N*-protected with the tetrachlorophthaloyl (TCP) group.

The synthesis of **4** started from commercially available D-galactosamine hydrochloride which was first converted to the 2-*N*-tetrachlorophthaloyl derivative followed by peracetylation with acetic anhydride to give fully protected galactose **1** as an anomeric mixture. The α and β anomers (3:7 molar ratio) were identified by NMR. To synthesize bromide **2**, we used TiBr_4_ as a bromination agent. Bromide **2** was obtained as a mixture of α and β anomers (1:4 molar ratio) and was used directly in the glycosylation. This mixture of **2** is chromatographically inseparable due to the highly reactive nature of the bromine group at the anomeric carbon, but, the anomers of **2** are readily distinguishable in the NMR spectrum (δ 6.65, d, *J*_1,2_ = 3.7 Hz for the α anomer and δ 6.35, d, *J*_1,2_ = 9.6 Hz for the β anomer).

**Figure 1:**
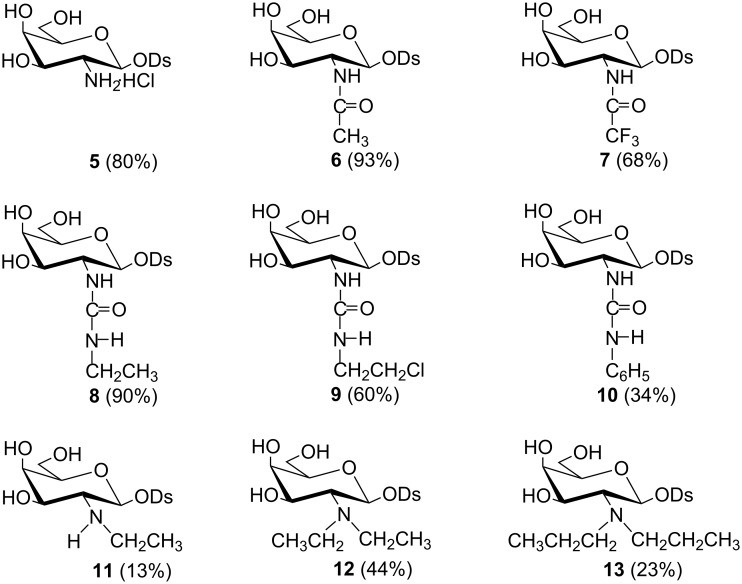
Derivatives of diosgenyl glycosides **5**–**13**.

Glycosylation of diosgenin with **2** was performed in dichloromethane by a “reverse” procedure: The glycosyl donor was added to the solution of diosgenin and the promoter (silver triflate) [31]. This procedure afforded the expected β glycoside **3** in 80% yield. The structure of **3** was determined by ^1^H and ^13^C NMR supported by COSY and HSQC techniques. The β linkage in **3**, as well as the ^4^*C*_1_ conformation of the pyranose ring were confirmed by the coupling constant of the anomeric proton (*J*_1,2_ = 8.4 Hz). The deprotection of **3** was achieved by using 98% hydrazine hydrate in refluxing ethanol. This procedure removes the TCP and acetyl protecting groups in a one-pot reaction and yields diosgenyl 2-amino-2-deoxy-β-D-galactopyranoside (**4**). Saponin **4** treated with the HCl in MeOH was converted into hydrochloride **5**. To explore the influence of different modifications of the amino group in **4** on its antimicrobial activity the *N*-acyl (**6**, **7**), 2-ureido (**8**–**10**), *N*-monoalkyl (**11**) and *N,N*-dialkyl (**12**, **13**) derivatives of diosgenyl 2-amino-2-deoxy-β-D-galactopyranoside were also synthesized (Figure 1).

The *N*-acetyl derivative **6** was obtained by the treatment of **4** with acetic anhydride in methanol with Et_3_N whereas *N*-trifluoroacetyl derivative **7** was obtained in a reaction of **4** with trifluoroacetic anhydride in pyridine. The IR, HRMS and NMR confirmed the structures of **6** and **7**. For example, the IR spectra of **6** and **7** show typical amide I and amide II bands at 1715–1650 cm^−1^. In turn, the ^13^C NMR spectrum of **7** is characterized by a quartet of the carbonyl carbon (≈157 ppm) with the *J*_C,F_ coupling constant ≈39 Hz and a quartet of the CF_3_ carbon (≈117 ppm) with the *J*_C,F_ coupling constant ≈290 Hz (see Supporting Information File 1 for experimental and NMR data).

Strategies for the preparation of ureido sugars usually involve the condensation of saccharides with ureas or the reaction of glycosylamines, amino sugar, or aminoglycosides with isocyanates, or their equivalents such as carbamates [32–33]. Ureido saponins presented here were obtained in the reaction of ethyl isocyanate (**8**), chloroethyl isocyanate (**9**) and phenyl isocyanate (**10**) with the amino group of **4**. Isocyanate was added to the 1:1 (v/v) chloroform–methanol solution of **4** and Et_3_N each time. The yields of these reactions were very good (90% for **8**) or good (60% for **9**). However, phenylurea derivative **10** was isolated with only 34% yield, and the formation of various byproducts was observed in this case. The structures of saponins **8**–**10** were established using IR, HRMS and NMR spectra. For example, the amide I and amide II bands at 1665–1550 cm^−1^ are visible in the IR spectra and the carbonyl carbon signals at 160–170 ppm are present in the ^13^C NMR spectra of **8**–**10** (see Supporting Information File 1 for experimental and NMR data).

To obtain the *N*-alkyl derivatives of **4**, a method called “two-step reductive alkylation of amines” was chosen. This method was successfully used to prepare *N*-alkyl derivatives of other amino sugars, including diosgenyl aminoglucosides [34–35]. The presented reductive alkylation consists of a reaction of amine **4** with the appropriate aldehyde, which results in the respective imine subsequently reduced with sodium cyanoborohydride to the alkylated amine. Thus, the reaction of **4** with 1.5 molar excess of acetaldehyde followed by the in situ reduction with NaBH_3_CN resulted in *N*-ethylamino (**11**) and *N*,*N*-diethylamino (**12**) saponins, which were separated by column chromatography. An analogous reaction of **4** with 3 molar excess of propionaldehyde, followed by the reduction with NaBH_3_CN resulted solely in *N*,*N*-dipropylaminosaponin **13**. The ^1^H and ^13^C NMR spectroscopy as well as the HRMS spectrometry confirmed the structures of **11**–**13** (see Supporting Information File 1 for experimental and NMR data).

### Evaluation of antimicrobial activity

The antimicrobial in vitro activities of **5**–**13** were tested against two fungal strains: *Candida albicans* and *Candida tropicalis*, and against the following six Gram-positive bacteria species: *Enterococcus faecalis*, *Enterococcus faecium*, *Staphylococcus aureus*, *Staphylococcus epidermidis*, *Streptococcus pyogenes* and *Streptococcus pneumoniae*. The minimum inhibitory concentrations (MICs) were determined by the broth microdilution method according to Clinical and Laboratory Standards Institute (CLSI) recommendations (for details see Supporting Information File 1). The MICs values are grouped in Table 1 and Table 2.

**Table 1:** Minimum inhibitory concentration (MIC) [μg/mL] for **5**–**13** against two fungi.

Compd.	*Candida albicans*	*Candida tropicalis*

**5**	64	4
**6**	16	8
**7**	>1024	>1024
**8**	>1024	64
**9**	64	16
**10**	>1024	>1024
**11**	8	4
**12**	4	2
**13**	8	8

**Table 2:** Minimum inhibitory concentration (MIC) [μg/mL] for **5**–**13** against Gram-positive bacteria.

Compd.	*E. faecalis*	*E. faecium*	*S. aureus* ATCC 25923	*S. aureus* ATCC 6538	*S. aureus* ATCC 6538/P	*S. epidermidis*	*S. pyogenes*	*S. pneumoniae*

**5**	>1024	>1024	>1024	>1024	>1024	>1024	>1024	>1024
**6**	16	8	16	32	32	8	8	32
**7**	32	>1024	>1024	>1024	>1024	>1024	>1024	>1024
**8**	512	16	>1024	>1024	>1024	>1024	>1024	>1024
**9**	16	8	16	16	32	8	8	8
**10**	>1024	>1024	>1024	>1024	>1024	>1024	>1024	>1024
**11**	8	2	4	4	8	2	2	2
**12**	16	>1024	>1024	16	64	32	8	32
**13**	64	>1024	>1024	64	128	32	64	32

The presented results indicate that some of the tested saponins are characterized by selective activity. Thus, the hydrochloride of **5** inhibits the growth of the fungus genus *Candida*; however, this saponin is more active against *C. tropicalis* (MIC 4 μg/mL) than against *C. albicans* (MIC 64 μg/mL). In turn, hydrochloride **5** has no inhibitory activity against the tested Gram-positive bacteria. The *N*-trifluoroacetyl derivative of diosgenyl 2-amino-2-deoxy-β-D-galactopyranoside **7** is active solely against *E. faecalis*. In contrast to **7**, the *N*-acetyl saponin **6** was found to inhibit the growth of all the tested fungi and bacteria with MICs of 8–32 μg/mL. Among the three ureido derivatives of **4**, solely diosgenyl 2-(2-chloroethylureido)-2-deoxy-β-D-galactopyranoside (**9**) exhibits a good activity against all the tested pathogens with MICs of 8–64 µg/mL, whereas **8** was effective against *C. tropicalis* and *E. faecium* only and compound **10** does not exhibit any antimicrobial activity. Among the presented saponins, *N*-ethyl derivative **11** exhibits the best antimicrobial activity against all the tested microorganisms with MIC values of 2–8 µg/mL. Both *N*,*N*-diethyl (**12**) and *N*,*N*-dipropyl (**13**) derivatives are active against both fungal and almost all the bacterial strains except *E. faecium* PCM 1859 and *S. aureus* ATCC 25923. These two strains of bacteria are completely resistant to **12** and **13**, whereas the growth of *E. faecalis* PCM 2673 as well as *S. aureus* ATCC 6538, and *S. aureus* ATCC 6538/P is inhibited by **12** and **13**.

The analysis of the relationship between structure and biological activity of the presented derivatives of diosgenyl 2-amino-2-deoxy-β-D-galactopyranoside (**4**) shows that *N*-alkylation of the amine function is the most advantageous way of modification. An introduction of one ethyl (**11**), two ethyl (**12**) or two propyl (**13**) groups at the amine function improves the antifungal properties of saponins **11–13** in comparison with hydrochloride **5** and the remaining compounds. In the case of antibacterial properties, *N*-ethyl derivative **11** is the most active among the all tested compounds. Moreover, derivative **11** acts against all tested Gram-positive bacteria. An introduction of an additional ethyl group worsens the antibacterial activity of **12** in comparison with **11**, particularly against *E*. *faecium* and *S*. *aureus* ATCC 25923. Two propyl groups cause that **13** exhibits even a weaker inhibitory effectivity than **12**. These findings are in full agreement with our previous findings concerning *N*-alkyl and *N*,*N*-dialkyl derivatives of diosgenyl 2-amino-2-deoxy-β-D-glucopyranoside [31]. Monoethyl derivatives of both glucosamine and galactosamine are the most active compounds against Gram-positive bacteria in the tested groups, regardless of the fact that hydrochloride of aminogalactoside **5** is completely inactive with respect to the tested bacteria whereas analogous hydrochloride of aminoglucoside is relatively active.

Transformation of the 2-amino group into a 2-ureido group rather negatively influences both antifungal and antibacterial activities of the presented saponins. Among the ureido compounds (**8**−**10**) only chloroethylureido derivative **9** exhibits weak inhibitory activity against tested Gram-positive bacteria. Its ethylureido analog **8** is inactive in respect of both fungi and bacteria, likewise phenylureido derivative **10**. It would seem that an electronegative Cl atom improves the inhibitory properties of ureido saponins against Gram-positive bacteria. However, the influence of the more electronegative three F atoms on an inhibitory effectivity of trifluoroacetyl derivative **7** is opposite. If *N*-acetyl derivative **6** is relatively active against tested fungi and bacteria, its *N*-trifluoroacetyl analog **7** does not act at all. The presented results show how complicated the relationships between structure and biological activity are.

## Conclusion

Diosgenyl 2-amino-2-deoxy-β-D-galactopyranoside, as well as its two *N*-acyl, three 2-ureido and three *N*-alkyl derivatives are reported. *N*-Alkylation of the amine function seems to be the most advantageous way of modification. Among the tested compounds, the *N*-alkyl derivatives are the most active against tested fungi and the *N*-ethyl derivative exhibits the best inhibitory activity against Gram-positive bacteria. The latter finding is in full agreement with our previous findings concerning the *N*-ethyl derivative of D-glucosamine. Apart from *N*-ethyl also *N*-acetyl and 2-chloroethylureido derivatives exhibit activity against Gram-positive bacteria. The change of D-glucosamine into D-galactosamine in diosgenyl 2-amino-2-deoxy-β-D-glycopyranoside impairs the antimicrobial properties of its hydrochloride.

## Supporting Information

File 1 Experimental procedures for the preparation of compounds **1**–**13**, spectroscopic data and information on the method of determination of the minimum inhibitory concentration.
