# Supplementary material for: Diosgenyl 2-amino-2-deoxy-β-D-galactopyranoside: synthesis, derivatives and antimicrobial activity
Source: Beilstein J Org Chem. 2017 Nov 1;13:2310–5. doi: 10.3762/bjoc.13.227 (PMC5687012; doi:10.3762/bjoc.13.227)
Supplement: File 1 — Experimental procedures for the preparation of compounds 1–13, spectroscopic data and information on the method of determination of the minimum inhibitory concentration. [file Beilstein_J_Org_Chem-13-2310-s001.pdf]

Supporting Information

for

Diosgenyl 2-amino-2-deoxy-β-D-galactopyranoside: synthesis, derivatives and antimicrobial activity

Henryk Myszka*[1], Patrycja Sokołowska[1], Agnieszka Cieślińska[1], Andrzej Nowacki[1], Maciej Jaśkiewicz[2], Wojciech Kamysz[2] and Beata Liberek[1]

Address: [1]Faculty of Chemistry, University of Gdańsk, Wita Stwosza 63, 80-308 Gdańsk, Poland and [2]Faculty of Pharmacy, Medical University of Gdańsk, Hallera 107, 80-416 Gdańsk, Poland

Email: Henryk Myszka* - henryk.myszka@ug.edu.pl

*Corresponding author

Experimental procedures for the preparation of compounds 1–13, spectroscopic data and information on the method of determination of the minimum inhibitory concentration

Contents

Experimental S2

Biological evaluation. materials and methods S8

NMR data for **3–13** S10

References S20

Experimental

Solvents and chemical reagents were purchased and used without further purification. The IR spectra were recorded as Nujol mulls or KBr pellets with a Bruker IFS 66 spectrophotometer. The ^{1}H and ^{13}C NMR spectra were recorded on a Varian Merkury 500 MHz instrument (500.13/125.76 MHz), using standard experimental conditions and $CDCl_3$, CD_3OD, pyridine-d_5 or a mixture of $CDCl_3$:CD_3OD (1:1, v/v) as solvents with internal Me_4Si. Structural assignments were based on the COSY and HSQC techniques. High-resolution mass spectra (HRMS) were marked on spectrometer Q-TOF UPLC/MS Agilent 6550 as monoisotopic masses. Positive-ion mode MALDI–TOF mass spectra were obtained using a Bruker Biflex III spectrometer with 4-cyano-4-hydroxycinnamic acid or 2,5-dihydroxybenzoic acid as matrixes. Thin-layer chromatography (TLC) was performed on aluminium plates coated with E. Merck Kieselgel 60 F_{254}. For the compounds detection the dry plates were heated at ca. 250 °C in a stream of hot air. Column chromatography (LC) was performed on MN Kieselgel 60 (<0.08 mm). For chromatography the following eluent systems (v/v) were used: A, 10:1 toluene/AcOEt; B, 4:1 CCl_4/acetone; C, 6:1 CCl_4/acetone; D, 7:1 $CHCl_3$/MeOH; E, 4:1 $CHCl_3$/MeOH.

1,3,4,6-Tetra-*O*-acetyl-2-deoxy-2-tetrachlorophthalimido-α,β-D-galactopyranose (1)

D-Galactosamine hydrochloride (4.32 g, 20 mmol) was added to a solution of 0.25 M NaOMe in methanol (80 mL) in small portions. After 15 min the opalescent solution was treated with 3,4,5,6-tetrachlorophthalic anhydride (TCPA, 3.57 g, 12 mmol). The mixture was vigorously stirred for ca. 20 min and then Et_3N (3.5 mL, 25 mmol) was added and a second portion of TCPA (3.57 g, 12 mmol). The solution was stirred at rt. for 24 h. Afterwards, methanol was evaporated under reduced pressure. The or-

ange syrup was dissolved in pyridine (70 mL), treated dropwise with acetic anhydride (35 mL) and stirred overnight at rt. Then, the solution was poured into ice-water, and the aqueous mixture was extracted with $CHCl_3$ (3 × 100 mL). The combined extracts were washed with water (100 mL), 5% aq. HCl (3 × 100 mL), saturated aq. $NaHCO_3$ (2 × 100 mL) and again water (100 mL). The organic layer was dried over Na_2SO_4, filtered and concentrated in vacuum. Column chromatography (solvent A) of the crude product yielded **1** (4.93 g, white solid, 41%) as an anomeric mixture: R_f 0.66 (solvent B); ^{1}H NMR (500 MHz, $CDCl_3$) (α): δ 6.44 (dd, 1 H, $J_{2,3}$ 12.0 Hz, $J_{3,4}$ 3.0 Hz H-3), 6.31 (d, 1 H, $J_{1,2}$ 3.2 Hz, H-1), 5.68 (d, 1 H, J 1.9 Hz, H-4), 4.91 (dd, 1 H, $J_{1,2}$ 3.2 Hz, $J_{2,3}$ 12.2 Hz, H-2), 4.51 (t, 1 H, J 6.6 Hz, H-5), 4.16 (m, 2 H, H-6, H-6'); (β): δ 6.44 (d, 1 H, $J_{1,2}$ 9.0 Hz, H-1), 5.86 (dd, 1 H, $J_{2,3}$ 11.7 Hz, $J_{3,4}$ 3.2 Hz, H-3), 5.51 (d, 1 H, $J_{3,4}$ 3.4 Hz, H-4), 4.20 (m, 3 H, H-5, H-6, H-6'); (α+β): 2.21, 2.19, 2.07, 2.07, 2.06, 2.04, 1.90, 1.89 (8 s, 24 H, 8 x OAc); ^{13}C NMR (100 MHz, $CDCl_3$) (α): δ 91.15 (C-1), 69.37 (C-5), 66.82 (C-4), 64.47 (C-3), 61.27 (C-6), 50.14 (C-2); (β): δ 90.03 (C-1), 71.78 (C-5), 67.56 (C-3), 66.26 (C-4), 61.09 (C-6), 51.05 (C-2); (α+β): δ 170.38, 170.35, 170.11, 170.07, 169.74, 169.68 169.67, 169.28 (8 x C=O in OAc), 140.74, 140.78 (2 x C=O in TCP), 130.09 (Ph), 20.97, 20.84, 20.69, 20.68, 20.64, 20.61, 20.55 (8 x CH_3).

3,4,6-Tri-*O*-acetyl-2-deoxy-2-tetrachlorophthalimido-α,β-D-galactopyranosyl bromide (2)

To a solution of **1** (1.0 g, 1.6 mmol) in dichloromethane (16 mL) and ethyl acetate (1.6 mL), $TiBr_4$ (1.24 g, 3.4 mmol) was added. The mixture was stirred at rt and monitored by TLC (solvent B). After 24 h second portion of $TiBr_4$ (1.17 g, 3.2 mmol) was added and the mixture was stirred again ca. 24 h, until TLC indicated the disappearance of **1**. The dark-red solution was diluted with $CHCl_3$ (80 mL), washed with H_2O (2

× 30 mL), saturated aq. $NaHCO_3$ (30 mL) and again H_2O (2 × 30 mL). The yellow organic layer was dried over Na_2SO_4 filtered and concentrated in vacuum. Compound **2** was obtained as an anomeric mixture (0.95 g, white foam, 93%) and was immediately used in the reaction with diosgenin. R_f 0.70 (solvent B); ^{1}H NMR (500 MHz, $CDCl_3$) (α): δ 6.65 (d, 1 H, $J_{1,2}$ 3.7 Hz H-1), 6.45 (dd, 1 H, $J_{2,3}$ 11.9 Hz, $J_{3,4}$ 3.1 Hz, H-3), 5.72 (d, 1 H, J 2.8 Hz, H-4), 4.82 (dd, 1 H, $J_{1,2}$ 3.6 Hz, $J_{2,3}$ 11.8 Hz, H-2), 4.58 (t, 1 H, J 6.6 Hz, H-5), 4.21 (m, 1 H, H-6) 4.16 (m, 1 H, H-6'); (β): δ 6.35 (d, 1 H, $J_{1,2}$ 9.6 Hz, H-1), 5.69 (dd, 1 H, $J_{2,3}$ 11.0 Hz, $J_{3,4}$ 3.2 Hz, H-3), 5.54 (d, 1 H, $J_{3,4}$ 3.4 Hz, H-4), 4.24 (m, 1 H, H-5), 4.22 (m, 1 H, H-6), 4.15 (m, 1 H, H-6'); (α+β): 2.23, 2.17, 2.08, 2.05, 1.91, 1.89 (6 s, 18 H, 6 x OAc); ^{13}C NMR (100 MHz, $CDCl_3$) (α): δ 87.84 (C-1), 71.44 (C-5), 66.29 (C-4), 65.59 (C-3), 60.83 (C-6), 53.42 (C-2); (β): δ 77.46 (C-1), 71.41 (C-5), 66.42 (C-3), 65.75 (C-4), 61.24 (C-6), 55.21 (C-2); (α+β): δ 170.38, 170.32, 169.79, 169.62, 168.92, 168.88 (6 x C=O in OAc), 140.72, 140.65 (2 x C=O in TCP), 126.75 (Ph), 20.83, 20.67, 20.65, 20.53, 20.50 (6 x CH_3).

Diosgenyl 3,4,6-tri-*O*-acetyl-2-deoxy-2-tetrachlorophthalimido-β-D-galactopyranoside (3)

A mixture of diosgenin (0.45 g, 1.1 mmol) and powdered 4 Å molecular sieves (3 g) in dichloromethane (40 mL) was stirred at rt for 10 min under nitrogen. Then *s*-collidine (0.6 mL) and silver triflate (0,86 g, 3,3 mmol) were added and the mixture was stirred under N_2 for the next 10 min. After this time the solution of bromide **2** (0.95 g, 1.5 mmol) in CH_2Cl_2 (15 mL) was added dropwise. The reaction mixture was stirred at rt overnight. After this time TLC (solvent C) showed the conversion of substrate into the products. The mixture was diluted with $CHCl_3$ (100 mL), filtered over a gel layer and concentrated under reduced pressure. The addition of methanol (100 mL) caused the precipitation of **3** (1.05 g, white powder, 80%): R_f 0.65 (solvent C); ^{1}H

and ^{13}C NMR data, see Table S1; MALDI-TOF-MS: m/z 970.2 $(M+H)^+$ and 992.3 $(M+Na)^+$.

Diosgenyl 2-amino-2-deoxy-β-D-galactopyranoside (4)

Glycoside **3** (0.4 g, 0.41 mmol) was dissolved in EtOH (8 mL) and then hydrazine hydrate (0,7 mL, 64–65% of N_2H_4) was added. After heating at 80 °C for 2 h the starting material was not observed in TLC (solvent D) and the solvents were evaporated in vacuum. The crude residue was treated with a small amount of methanol to cause the precipitation of **4** (0.21 g, white powder, 89%): R_f 0.18 (solvent D); ^1H and ^{13}C NMR data, see Table S2; MALDI-TOF-MS: m/z 575.0 $(M)^+$.

Diosgenyl 2-amino-2-deoxy-β-D-galactopyranoside hydrochloride (5)

Saponin **4** (0.21 g, 0.36 mmol) was dissolved in $CHCl_3$/MeOH (1:1, v/v) and a stoichiometric amount of 1.3% HCl in MeOH (1.26 mL) was added. The hydrochloride was precipitated with ethyl ether to give **5** (0.2 g, amorphous powder, 80%): R_f 0.18 (solvent D); ^1H and ^{13}C NMR data, see Table S3; HRMS (ESI) m/z: $(M\text{-}Cl)^+$ calcd: 576.3900; found: 576.3892.

Diosgenyl 2-acetamido-2-deoxy-β-D-galactopyranoside (6)

The suspension of **4** (0.1 g, 0.17 mmol) in MeOH (10 mL) and Et_3N (0.17 mL) was treated with Ac_2O (1 mL). It was stirred at rt for 1 h until TLC (solvent D) showed the complete conversion of **4**. Then, the solvents were evaporated in vacuum to give **6** (97 mg, semisolid, 93%): R_f 0.27 (solvent D); IR: v 3227 (N–H), 1657 (amide I), 1599 cm^{-1} (amide II); ^1H and ^{13}C NMR data, see Table S4; HRMS (ESI) m/z: $(M+H)^+$ calcd: 618.4006; found: 618.3998.

Diosgenyl 2-deoxy-2-trifluoroacetamido-β-D-galactopyranoside (7)

To a solution of **4** (81 mg, 0.14 mmol) in pyridine (1.2 mL), cooled in an ice-water bath, trifluoroacetic anhydride (0.2 mL) in diethyl ether (1 mL) was added under vigorous stirring. Then, the mixture was warmed slowly to rt. After being stirred for 24 h, the mixture was diluted with ethyl acetate (20 mL), and then the organic phase was washed with water (2 × 7 mL), dried over $MgSO_4$ and concentrated. The crude residue was purified by column chromatography (eluent D) to give **7** (64 mg, colourless amorphous solid, 68%): R_f 0.24 (solvent D); IR: v 3634 (N–H), 1714 (amide I), 1565 cm^{-1} (amide II); ^{1}H and ^{13}C NMR data, see Table S5; HRMS (ESI) m/z: $(M+H)^+$ calcd: 672.3723; found: 672.3715.

General procedure for the synthesis of ureido derivatives 8–10

To a solution of amino glycoside **4** (0.1 g, 0.17 mmol) in $CHCl_3$/MeOH (1:1, v/v; 20 mL) Et_3N (0.1 mL) and the appropriate isocyanate (0.7 mmol) were added. The mixture was stirred vigorously at rt until TLC (solvent D) showed the complete conversion into the ureido product (ca. 1 h). Then, the solvents were evaporated to dryness and the crude residue was dissolved in $CHCl_3$/MeOH (1:1, v/v; 2 mL). Addition of petroleum ether (dropwise ca. 2 mL) afforded the ureido product as a white solid, which was separated on a centrifuge to give the expected product.

Diosgenyl 2-deoxy-2-ethylureido-β-D-galactopyranoside (8)

Reaction of **4** with ethyl isocyanate (56 μL) gave **8** (0.11 g, white powder, 90%): R_f 0.44 (solvent D); IR (KBr): v 3299 (N–H), 1664 (amide I), 1563 cm^{-1} (amide II); ^{1}H and ^{13}C NMR data, see Table S6; HRMS (ESI) m/z: $(M+H)^+$ calcd: 647.4271; found: 647.4267.

Diosgenyl 2-(2-chloroethylureido)-2-deoxy-β-D-galactopyranoside (9)

Reaction of **4** with 2-chloroethyl isocyanate (60 µL) yielded **9** (0.07 g, white semisolid, 60%): R_f 0.40 (solvent D); IR: v 3329 (N–H), 1663 (amide I), 1558 cm^{-1} (amide II); ^{1}H and ^{13}C NMR data, see Table S7; HRMS (ESI) m/z: $(M+H)^+$ calcd: 681.3882; found: 681.3878.

Diosgenyl 2-deoxy-2-phenylureido-β-D-galactopyranoside (10)

Reaction of **4** with phenyl isocyanate (76 µL) led to **10** (0.04 g, white solid, 34%): R_f 0.49 (solvent D); IR: v 3352 (N–H), 1670 (amide I), 1555 cm^{-1} (amide II); ^{1}H and ^{13}C NMR data, see Table S8; HRMS (ESI) m/z: $(M+H)^+$ calcd: 695.4271; found: 695.4263.

Diosgenyl 2-deoxy-2-ethylamino- (11) and 2-deoxy-2-diethylamino-β-D-galactopyranoside (12)

Acetaldehyde (30 µL, 0.52 mmol) was added to a solution of **4** (0.2 g, 0.35 mmol) in $CHCl_3$/MeOH (1:1, v/v; 5 mL). The mixture was stirred at rt for 0.5 h. Then, the first portion of $NaBH_3CN$ (30 mg, 0.48 mmol) was added and stirring was continued for 24 h. After this time the second portion of $NaBH_3CN$ (30 mg, 0.48 mmol) was added and stirring was continued for the next 24 h. The end of reaction was detected by TLC (solvent D). Then, the solvents were evaporated under reduced pressure and the residue was separated by column chromatography (solvent D) to give two products. The first was **11** (28 mg, colourless solid, 13%): R_f 0.29 (solvent D); ^{1}H and ^{13}C NMR data, see Table S9; HRMS (ESI) m/z: $(M+H)^+$ calcd: 604.4213; found: 604.4207.

The second was **12** (98 mg, colourless solid, 44%), R_f 0.59 (solvent D); ^{1}H and ^{13}C NMR data, see Table S10; HRMS (ESI) m/z: $(M+H)^+$ calcd: 632.4526; found: 632.4519.

Diosgenyl 2-deoxy-2-dipropylamino-β-D-galactopyranoside (13)

Propionaldehyde (76 µL, 1.06 mmol) was added to a solution of **4** (0.2 g, 0.35 mmol) in $CHCl_3$/MeOH (1:1, v/v; 8 mL). The mixture was stirred at rt for 0.5 h. Then, $NaBH_3CN$ (66 mg, 1.05 mmol) was added and stirring was continued for 48 h. The end of reaction was detected by TLC (solvent E). Then, the solvents were evaporated under reduced pressure and the residue was chromatographed (solvent E) to yield **13** (54 mg, white solid, 23%); R_f 0.61 (solvent E); ^{1}H and ^{13}C NMR data, see Table 11; HRMS (ESI) m/z: $(M+H)^+$ calcd: 660.4839; found: 660.4829.

Biological evaluation, materials and methods

Reference strains of bacteria: *Enterococcus faecalis* PCM 2673, *E. faecium* PCM 1859, *Staphylococcus aureus* ATCC 25923, *S. aureus* ATCC 6538, *S. aureus* ATCC 6538/P, *S. epidermidis* ATCC 14490, *Streptococcus pyogenes* PCM 465, *S. pneumoniae* ATCC and fungi: *Candida albicans* ATCC 10231, *C. tropicalis* PCM 2681 used in the study were purchased from the Polish Collection of Microorganisms (Polish Academy of Sciences, Wroclaw, Poland). Before the tests, bacterial strains were inoculated in Mueller–Hinton broth (Biocorp, Poland) for 24 h and incubated at 37 °C with 150 rpm shaking, whereas fungi were inoculated in Sabouraud broth (Biocorp, Poland) and cultured for 48 h at 25 °C with 150 rpm shaking. The minimum inhibitory concentrations (MICs) of tested compounds were determined according to Clinical and Laboratory Standards Institute (CLSI) protocol [1,2]. The experiments were conducted on 96-well microtiter plates, with final volume of 100 µL which contained positive control (growth control – microorganisms without tested compound) and negative control (sterility control – only microbiological broth). Bacteria at initial inoculums of 5×10^5 CFU/mL in Mueller–Hinton Broth and fungi at initial inoculums of

10^3 CFU/mL in Sabouraud broth were exposed to the increasing concentrations of the compounds (1–1024 μg/mL). Plates for bacteria were incubated at 37 °C for 18 h while the ones for fungi were incubated at 25 °C. The MIC was taken as the lowest concentration at which a noticeable growth of microorganisms was inhibited. The experiments were conducted in triplicate.

NMR data for compounds 3–13

Table S1: ^{1}H and ^{13}C NMR data for glycoside **3** ($CDCl_3$).*

Proton	δ (ppm) and *J* (Hz)	Carbon	δ (ppm)
H-1’	5.34 (d, 1 H, $J_{1,2}$ 8.4)	C-1’	96.93
H-2’	4.46 (dd, 1 H, $J_{1,2}$ 8.4, $J_{2,3}$ 11.3)	C-2’	52.44
H-3’	5.60 (dd, 1 H, $J_{2,3}$ 11.3, $J_{3,4}$ 3.1)	C-3’	68.21
H-4’	5.39 (d, 1 H, $J_{3,4}$ 3.1)	C-4’	66.85
H-5’	3.96 (t, 1 H, $J_{5,6}$ 6.7)	C-5’	70.78
H-6a’	4.09 (dd, 1 H $J_{5,6'}$ 6.7)	C-6’	61.28
H-6b’	4.16 (dd, 1 H, $J_{6a',6b'}$ 10.9)		
CH_3 (OAc)	1.81 (s), 1.99 (s), 2.12 (s)	CH_3 (OAc)	20.60, 20.71, 20.80
		C=O (OAc)	169.91, 170.31, 170.38
		C=O (TCP)	140.74, 141.45
		Ph	130.03
H-1	0.98 (m, 1 H), 1.73 (m, 1 H)	C-1	37.07
H-2	1.38 (m, 1 H), 1.55 (m, 1 H)	C-2	28.80
H-3	3.42 (m, 1 H)	C-3	79.73
H-4	1.87 (m, 1 H), 1.91 (m, 1 H)	C-4	38.68
		C-5	139.97
H-6	5.20 (bs, 1 H)	C-6	122.09
H-7	1.44 (m, 1 H), 1.89 (m, 1 H)	C-7	31.38
H-8	1.55 (m, 1 H)	C-8	30.29
H-9	0.82 (m, 1 H)	C-9	49.95
		C-10	36.74
H-11	1.41 (m, 2 H)	C-11	20.67
H-12	1.10 (m, 1 H), 1.63 (m, 1 H)	C-12	39.70
		C-13	40.24
H-14	1.03 (m, 1 H)	C-14	56.42
H-15	1.19 (m, 1 H), 1.92 (m, 1 H)	C-15	32.01
H-16	4.32 (q, 1 H, *J* 7.5)	C-16	80.78
H-17	1.68 (m, 1 H)	C-17	62.07
H-18	0.69 (s, CH_3)	C-18	16.25
H-19	0.86 (s, CH_3)	C-19	19.34
H-20	1.78 (m, 1 H)	C-20	41.60
H-21	0.89 (d, CH_3, *J* 6.9)	C-21	14.51
		C-22	109.28
H-23	1.60 (m, 2 H)	C-23	31.82
H-24	1.47 (m, 1 H), 1.81 (m, 1 H)	C-24	29.40
H-25	1.51 (m, 1 H)	C-25	31.36
H-26	3.30 (t, 1 H, $J_{26,25}$ 11.0),<br>3.39 (dd, 1 H, $J_{26,25}$ 5.0, *J* 10.7)	C-26	66.61
H-27	0.71 (d, CH_3, *J* 6.3)	C-27	17.13

* Atoms with apostrophe belong to D-galactosamine residue.

Table S2: ^{1}H and ^{13}C NMR data for glycoside **4** ($CDCl_3$:CD_3OD = 1:1).

Proton	δ (ppm) and *J* (Hz)	Carbon	δ (ppm)
H-1’	4.30 (d, 1 H, $J_{1,2}$ 8.1)	C-1’	101.45
H-2’	2.90 (dd, 1 H, $J_{1,2}$ 8.4, $J_{2,3}$ 10.0)	C-2’	53.35
H-3’	3.40 (dd, 1 H, $J_{2,3}$ 11.3, $J_{3,4}$ 3.1)	C-3’	72.97
H-4’	3.79 (d, 1 H, $J_{3,4}$ 3.1)	C-4’	67.96
H-5’	3.47 (t, 1 H, $J_{3,4}$ 3.1)	C-5’	75.06
H-6’	3.74 (d, 2 H, $J_{5,6a}$ 6.1, $J_{5,6e}$ 6.2)	C-6’	61.07
H-1	1.06 (m, 1 H), 1.83 (m, 1 H)	C-1	37.12
H-2	1.42 (m, 1 H), 1.63 (m, 1 H)	C-2	28.52
H-3	3.58 (m, 1 H)	C-3	78.40
H-4	2.21 (t, 1 H, *J* 11.9),<br>2.39 (dd, 1 H, *J* 13.1)	C-4	38.55
		C-5	140.32
H-6	5.35 (d, 1 H, *J* 4.4)	C-6	121.53
H-7	1.52 (m, 1 H), 1.98 (m, 1 H)	C-7	31.91
H-8	1.60 (m, 1 H)	C-8	30.06
H-9	0.92 (m, 1 H)	C-9	50.11
		C-10	36.69
H-11	1.51 (m, 2 H)	C-11	20.66
H-12	1.16 (m, 1 H), 1.73 (m, 1 H)	C-12	39.59
		C-13	40.15
H-14	1.11 (m, 1 H)	C-14	56.42
H-15	1.28 (m, 1 H), 1.96 (m, 1 H)	C-15	31.49
H-16	4.39 (q, 1 H, *J* 7.5)	C-16	80.91
H-17	1.74 (m, 1 H)	C-17	62.05
H-18	0.78 (s, CH_3)	C-18	15.86
H-19	1.02 (s, CH_3)	C-19	18.92
H-20	1.87 (m, 1 H)	C-20	41.54
H-21	0.95 (d, CH_3, *J* 6.9)	C-21	13.93
		C-22	109.46
H-23	1.68 (m, 2 H)	C-23	31.12
H-24	1.56 (m, 1 H), 1.92 (m, 1 H)	C-24	29.45
H-25	1.63 (m, 1 H)	C-25	31.36
H-26	3.34 (m, 1 H), 3.44 (m, 1 H)	C-26	66.69
H-27	0.71 (d, CH_3, *J* 6.3)	C-27	16.56

Table S3: ^{1}H and ^{13}C NMR data for glycoside **5** (CD_3OD).

Proton	δ (ppm) and *J* (Hz)	Carbon	δ (ppm)
H-1’	4.63 (d, 1 H, $J_{1,2}$ 8.3)	C-1’	97.49
H-2’	3.11 (dd, 1 H, $J_{1,2}$ 8.4, $J_{2,3}$ 10.6)	C-2’	53.67
H-3’	3.65 (dd, 1 H, $J_{2,3}$ 10.7, $J_{3,4}$ 3.4)	C-3’	70.02
H-4’	3.87 (d, 1 H, $J_{3,4}$ 2.6)	C-4’	67.80
H-5’	3.56 (t, 1 H, $J_{5,6}$ 6.0)	C-5’	75.60
H-6’	3.75 (m, 2 H, $J_{5,6}$ 4.0)	C-6’	60.80
H-1	1.09 (m, 1 H), 1.92 (m, 1 H)	C-1	37.00
H-2	1.42 (m, 1 H), 1.62 (m, 1 H)	C-2	28.47
H-3	3.66 (m, 1 H)	C-3	78.39
H-4	2.31 (t, 1 H, *J* 11.3), 2.44 (dd, 1 H, *J* 13.0)	C-4	37.98
		C-5	140.24
H-6	5.40 (d, 1 H, *J* 4.7)	C-6	121.52
H-7	1.58 (m, 1 H), 2.01 (m, 1 H)	C-7	31.77
H-8	1.62 (m, 1 H)	C-8	30.03
H-9	0.98 (m, 1 H)	C-9	50.23
		C-10	36.57
H-11	1.56 (m, 2 H)	C-11	20.57
H-12	1.22 (m, 1 H), 1.76 (m, 1 H)	C-12	39.48
		C-13	40.01
H-14	1.15 (m, 1 H)	C-14	56.34
H-15	1.29 (m, 1 H), 1.99 (m, 1 H)	C-15	31.38
H-16	4.40 (q, 1 H, *J* 7.3)	C-16	80.79
H-17	1.74 (m, 1 H)	C-17	62.32
H-18	0.82 (s, CH_3)	C-18	15.35
H-19	1.06 (s, CH_3)	C-19	18.40
H-20	1.90 (m, 1 H)	C-20	41.50
H-21	0.96 (d, CH_3, *J* 7.0)	C-21	13.46
		C-22	109.19
H-23	1.71 (m, 2 H)	C-23	31.01
H-24	1.66 (m, 1 H), 1.96 (m, 1 H)	C-24	29.21
H-25	1.66 (m, 1 H)	C-25	31.32
H-26	3.34 (t, 1 H, $J_{26,25}$ 8.9), 3.45 (dd, 1 H, $J_{26,25}$ 4.0)	C-26	66.45
H-27	0.79 (d, CH_3, *J* 6.3)	C-27	16.09

Table S4: ^{1}H and ^{13}C NMR data for glycoside **6** ($CDCl_3$:CD_3OD = 1:1).

Proton	δ (ppm) and *J* (Hz)	Carbon	δ (ppm)
H-1’	4.51 (d, 1 H, $J_{1,2}$ 8.4)	C-1’	99.81
H-2’	3.73 (m, 1 H)	C-2’	53.65
H-3’	3.62 (m, 1 H)	C-3’	71.78
H-4’	3.83 (m, 1 H)	C-4’	68.24
H-5’	3.75 (m, 1 H)	C-5’	74.78
H-6’	3.75 (m, 1 H), 3.62 (m, 1 H)	C-6’	61.11
CH_3 (Ac)	1.98 (s)	CH_3 (Ac)	19.70
		C=O (Ac)	170.52
H-1	1.04 (m, 1 H), 1.84 (m, 1 H)	C-1	37.13
H-2	1.42 (m, 1 H), 1.62 (m, 1 H)	C-2	28.52
H-3	3.50 (m, 1 H)	C-3	78.86
H-4	2.13 (m, 1 H, *J* 12.8),<br>2.30 (m, 1 H, *J* 12.2)	C-4	38.69
		C-5	140.50
H-6	5.32 (bs, 1 H)	C-6	121.43
H-7	1.54 (m, 1 H), 1.96 (m, 1 H)	C-7	31.92
H-8	1.61 (m, 1 H)	C-8	30.07
H-9	0.93 (m, 1 H)	C-9	50.10
		C-10	36.71
H-11	1.51 (m, 2 H)	C-11	20.70
H-12	1.18 (m, 1 H), 1.75 (m, 1 H)	C-12	39.60
		C-13	40.17
H-14	1.12 (m, 1 H)	C-14	56.42
H-15	1.28 (m, 1 H), 1.97 (m, 1 H)	C-15	31.51
H-16	4.39 (q, 1 H, *J* 7.4)	C-16	80.94
H-17	1.75 (m, 1 H)	C-17	62.00
H-18	0.78 (s, CH_3)	C-18	15.92
H-19	0.99 (s, CH_3)	C-19	18.98
H-20	1.87 (m, 1 H)	C-20	41.55
H-21	0.94 (d, CH_3, *J* 6.8)	C-21	13.98
		C-22	109.51
H-23	1.59 (m, 1 H), 1.69 (m, 1 H)	C-23	31.13
H-24	1.56 (m, 1 H), 1.88 (m, 1 H)	C-24	29.32
H-25	1.63 (m, 1 H)	C-25	31.35
H-26	3.34 (m, 1 H), 3.45 (m, 1 H)	C-26	66.72
H-27	0.79 (d, CH_3, *J* 6.3)	C-27	16.62

Table S5: ^{1}H and ^{13}C NMR data for glycoside **7** ($CDCl_3$:CD_3OD = 1:1).

Proton	δ (ppm) and *J* (Hz)	Carbon	δ (ppm)
H-1'	4.51 (d, 1 H, $J_{1,2}$ 8.4)	C-1'	99.45
H-2'	3.87 (m, 1 H)	C-2'	53.80
H-3'	3.66 (m, 1 H, $J_{2,3}$ 10.8, $J_{3,4}$ 2.9)	C-3'	70.72
H-4'	3.83 (m, 1 H)	C-4'	68.20
H-5'	3.73 (m, 1 H)	C-5'	74.84
H-6'	3.74 (m, 2 H)	C-6'	60.78
NH	4.56 (d, $J_{NH,2}$ 8.4)		
		CF_3	117.24 (q, $J_{C,F}$ ~290 Hz)
		C=O	157.26 (q, $J_{C,F}$ ~39 Hz)
H-1	1.02 (m, 1 H), 1.85 (m, 1 H)	C-1	37.10
H-2	1.41 (m, 1 H), 1.61 (m, 1 H)	C-2	28.51
H-3	3.47 (m, 1 H)	C-3	79.05
H-4	2.07 (m, 1 H, *J* 14.5), 2.27 (m, 1 H, *J* 13.5)	C-4	38.53
		C-5	140.39
H-6	5.31 (bs, 1 H)	C-6	121.22
H-7	1.56 (m, 1 H), 2.06 (m, 1 H)	C-7	31.51
H-8	1.61 (m, 1 H)	C-8	30.06
H-9	0.93 (m, 1 H)	C-9	50.10
		C-10	36.68
H-11	1.47 (m, 2 H)	C-11	20.69
H-12	1.18 (m, 1 H), 1.74 (m, 1 H)	C-12	39.80
		C-13	40.16
H-14	1.12 (m, 1 H)	C-14	56.42
H-15	1.27 (m, 1 H), 1.96 (m, 1 H)	C-15	31.92
H-16	4.38 (q, 1 H, *J* 7.4)	C-16	80.93
H-17	1.76 (m, 1 H)	C-17	62.00
H-18	0.79 (s, CH_3)	C-18	15.91
H-19	0.98 (s, CH_3)	C-19	18.92
H-20	1.87 (m, 1 H)	C-20	41.54
H-21	0.94 (d, CH_3, *J* 6.8)	C-21	13.96
		C-22	109.51
H-23	1.55 (m, 1 H), 1.68 (m, 1 H)	C-23	31.12
H-24	1.49 (m, 1 H), 1.87 (m, 1 H)	C-24	29.11
H-25	1.61 (m, 1 H)	C-25	31.35
H-26	3.34 (m, 1 H), 3.44 (m, 1 H)	C-26	66.71
H-27	0.77 (d, CH_3)	C-27	16.61

Table S6: ^{1}H and ^{13}C NMR data for glycoside **8** (Pyridine-d_5).

Proton	δ (ppm) and *J* (Hz)	Carbon	δ (ppm)
H-1’	5.04 (d, 1 H, $J_{1,2}$ 8.1)	C-1’	102.42
H-2’	4.42 (m, 1 H, $J_{1,2}$ = $J_{2,3}$ 8.4)	C-2’	56.82
H-3’	4.31 (dd, 1 H, , $J_{2,3}$ 9.9, $J_{3,4}$ 2.5)	C-3’	75.16
H-4’	4.56 (m, 1 H)	C-4’	69.88
H-5’	4.07 (t, 1 H, *J* 5.8)	C-5’	77.32
H-6’	4.47 (m, 2 H)	C-6’	62.81
NH	6.45 (d, $J_{NH,2}$ 5.8)		
–CH_2–	3.31 (m, 1 H), 3.41 (m, 1 H)	–CH_2–	35.71
–CH_3	1.06 t (J = 7.1)	–CH_3	16.17
		C=O	161.44
H-1	0.95 (m, 1 H), 1.67 (m, 1 H)	C-1	37.33
H-2	1.71 (m, 1 H), 2.11 (m, 1 H)	C-2	30.53
H-3	3.89 (m, 1 H)	C-3	77.32
H-4	2.35 (dd, 1 H, *J* 1.9, *J* 12.7), 2.59 (t, 1 H, *J* 11.8)	C-4	39.50
		C-5	141.10
H-6	5.27 (bs, 1 H)	C-6	122.15
H-7	1.48 (m, 1 H), 1.88 (m, 1 H)	C-7	31.99
H-8	1.59 (m, 1 H)	C-8	30.96
H-9	0.89 (m, 1 H)	C-9	50.59
		C-10	37.72
H-11	1.41 (m, 2 H)	C-11	21.44
H-12	1.12 (dd, 1 H, *J* 5.5, *J* 12.2), 1.71 (m, 1 H)	C-12	40.81
		C-13	40.21
H-14	1.12 (m, 1 H, *J* 5.5, *J* 11.6)	C-14	57.01
H-15	1.44 (m, 1 H), 2.04 (m, 1 H, *J* 5.8)	C-15	32.60
H-16	4.56 (m, 1 H, *J* 3.7)	C-16	81.45
H-17	1.82 (t, 1 H, *J* 7.4)	C-17	63.23
H-18	0.85 (s, CH_3)	C-18	16.72
H-19	0.92 (s, CH_3)	C-19	19.76
H-20	1.97 (m, 1 H, *J* 6.6)	C-20	42.32
H-21	1.16 (d, CH_3, *J* 6.9)	C-21	15.39
		C-22	109.63
H-23	1.70 (m, 2 H)	C-23	32.54
H-24	1.58 (m, 2 H)	C-24	29.61
H-25	1.54 (m, 1 H)	C-25	32.16
H-26	3.52 (m, 1 H), 3.60 (m, 1 H, *J* 11.6)	C-26	67.22
H-27	0.71 (d, CH_3, *J* 5.4)	C-27	17.68

Table S7: ^{1}H and ^{13}C NMR data for glycoside **9** (Pyridine-d_5).

Proton	δ (ppm) and *J* (Hz)	Carbon	δ (ppm)
H-1’	5.08 (d, 1 H, $J_{1,2}$ 8.5)	C-1’	101.99
H-2’	4.46 (m, 1 H)	C-2’	56.80
H-3’	4.36 (d, 1 H, *J* 8.0)	C-3’	74.62
H-4’	4.56 (m, 1 H)	C-4’	69.98
H-5’	4.08 (m, 1 H)	C-5’	77.28
H-6’	4.47 (m, 2 H)	C-6’	62.78
NH	6.89 (d, $J_{NH,2}$ 4.9)		
–CH_2–	3.63 (m, 1 H), 3.73 (m, 1 H)	–CH_2–	43.09
–CH_2Cl	3.72 (m, 1 H), 3.78 (m, 1 H)	–CH_2Cl	45.49
		C=O	160.98
H-1	0.94 (m, 1 H), 1.66 (m, 1 H)	C-1	37.37
H-2	1.75 (m, 1 H), 2.11 (m, 1 H)	C-2	30.53
H-3	3.90 (m, 1 H)	C-3	78.62
H-4	2.44 (dd, 1 H, *J* 1.5, *J* 12.2), 2.64 (t, 1 H, *J* 11.9)	C-4	39.59
		C-5	141.23
H-6	5.29 (m, 1 H)	C-6	122.09
H-7	1.45 (m, 1 H), 1.87 (m, 1 H)	C-7	31.99
H-8	1.58 (m, 1 H)	C-8	30.96
H-9	0.90 (m, 1 H)	C-9	50.61
		C-10	37.77
H-11	1.42 (m, 2 H)	C-11	21.45
H-12	1.10 (dd, 1 H, *J* 5.1, *J* 10.9), 1.74 (m, 1 H)	C-12	40.81
		C-13	40.23
H-14	1.08 (m, 1 H)	C-14	57.02
H-15	1.42 (m, 1 H), 2.02 (m, 1 H)	C-15	32.62
H-16	4.56 (m, 1 H)	C-16	81.45
H-17	1.82 (t, 1 H, *J* 7.1)	C-17	63.23
H-18	0.85 (s, CH_3)	C-18	16.72
H-19	0.94 (s, CH_3)	C-19	19.80
H-20	1.99 (m, 1 H)	C-20	42.32
H-21	1.15 (d, CH_3, *J* 6.0)	C-21	15.39
		C-22	109.63
H-23	1.68 (m, 2 H)	C-23	32.54
H-24	1.58 (m, 2 H)	C-24	29.62
H-25	1.54 (m, 1 H)	C-25	32.16
H-26	3.52 (m, 1 H), 3.59 (m, 1 H)	C-26	67.22
H-27	0.70 (d, CH_3)	C-27	17.68

Table S8: ^{1}H and ^{13}C NMR data for glycoside **10** ($CDCl_3$:CD_3OD = 1:1).

Proton	δ (ppm) and *J* (Hz)	Carbon	δ (ppm)
H-1’	4.50 (d, 1 H, $J_{1,2}$ 8.3)	C-1’	100.40
H-2’	3.56 (m, 1 H)	C-2’	54.81
H-3’	3.64 (dd, 1 H, $J_{2,3}$ 10.0, $J_{3,4}$ 2.6)	C-3’	72.25
H-4’	3.87 (m, 1 H)	C-4’	68.20
H-5’	3.48 (m, 1 H)	C-5’	74.64
H-6’	3.76 (m, 2 H)	C-6’	61.18
Ph	6.98 (t, 1 H), 7.24 (d, 2 H), 7.30 (d, 2 H)	Ph	119.49, 122.61, 128.78
		C=O	170.74
H-1	1.02 (m, 1 H), 1.85 (m, 1 H)	C-1	37.10
H-2	1.58 (m, 1 H), 1.92 (m, 1 H)	C-2	29.43
H-3	3.58 (m, 1 H)	C-3	78.97
H-4	2.18 (m, 1 H), 2.33 (m, 1 H, *J* 11.8)	C-4	38.54
		C-5	140.26
H-6	5.28 (m, 1 H)	C-6	121.66
H-7	1.51 (m, 1 H, *J* 6.2, J_{gem} 10.2), 1.93 (dd, 1 H, *J* 6.0, J_{gem} 12.0)	C-7	31.94
H-8	1.62 (m, 1 H)	C-8	30.09
H-9	1.00 (m, 1 H)	C-9	50.03
		C-10	36.73
H-11	1.48 (m, 2 H)	C-11	20.72
H-12	1.15 (m, 1 H), 1.70 (m, 1 H)	C-12	39.62
		C-13	40.19
H-14	1.12 (m, 1 H)	C-14	56.39
H-15	1.24 (dd, 1 H, *J* 6.2, J_{gem} 12.5), 1.96 (dd, 1 H, *J* 6.0, J_{gem} 12.5)	C-15	31.55
H-16	4.38 (q, 1 H, *J* 7.8)	C-16	80.94
H-17	1.74 (m, 1 H)	C-17	61.93
H-18	0.75 (s, CH_3)	C-18	16.04
H-19	0.95 (s, CH_3)	C-19	19.08
H-20	1.84 (m, 1 H)	C-20	41.55
H-21	0.93 (d, CH_3, *J* 7.0)	C-21	14.12
		C-22	109.56
H-23	1.60 (m, 1 H), 1.66 (m, 1 H)	C-23	31.17
H-24	1.41 (m, 1 H), 1.61 (m, 1 H)	C-24	28.54
H-25	1.58 (m, 1 H)	C-25	31.32
H-26	3.33 (m, 1 H), 3.44 (m, 1 H)	C-26	66.79
H-27	0.76 (d, CH_3, *J* 6.2)	C-27	16.77

Table S9: ^{1}H and ^{13}C NMR data for glycoside **11** ($CDCl_3$).

Proton	δ (ppm) and *J* (Hz)	Carbon	δ (ppm)
H-1’	4.89 (d, 1 H, $J_{1,2}$ 8.1)	C-1’	98.29
H-2’	3.16 (m, 1 H)	C-2’	58.97
H-3’	3.99 (dd, 1 H, $J_{2,3}$ 10.1, $J_{3,4}$ 4.2)	C-3’	69.17
H-4’	4.18 (m, 1 H)	C-4’	68.08
H-5’	3.70 (m, 1 H)	C-5’	73.81
H-6’	3.87 (m, 2 H)	C-6’	61.21
$–CH_2–$	2.26 (m, 1 H), 2.45 (m, 1 H)	$–CH_2–$	38.61
$–CH_3$	1.38 (m, 3 H)	$–CH_3$	12.41
H-1	1.07 (m, 1 H), 1.86 (m, 1 H)	C-1	37.11
H-2	1.55 (m, 1 H), 1.59 (m, 1 H)	C-2	31.77
H-3	3.64 (m, 1 H)	C-3	79.58
H-4	2.29 (m, 2 H)	C-4	38.63
		C-5	139.66
H-6	5.42 (m, 1 H, *J* 4.7)	C-6	122.64
H-7	1.30 (m, 1 H), 2.01 (m, 1 H)	C-7	32.09
H-8	1.62 (m, 1 H)	C-8	30.31
H-9	0.95 (m, 1 H)	C-9	50.00
		C-10	36.76
H-11	1.49 (m, 2 H)	C-11	20.88
H-12	1.18 (m, 1 H), 1.76 (m, 1 H)	C-12	39.75
		C-13	40.27
H-14	1.10 (m, 1 H)	C-14	56.46
H-15	1.27 (m, 1 H), 1.98 (m, 1 H)	C-15	31.85
H-16	4.41 (q, 1 H, *J* 7.8)	C-16	80.78
H-17	1.76 (m, 1 H)	C-17	62.16
H-18	0.80 (s, CH_3)	C-18	16.32
H-19	1.02 (s, CH_3)	C-19	19.39
H-20	1.87 (m, 1 H)	C-20	41.62
H-21	0.98 (d, CH_3, *J* 6.7)	C-21	14.57
		C-22	109.25
H-23	1.60 (m, 1 H), 1.69 (m, 1 H)	C-23	29.80
H-24	1.48 (m, 1 H), 1.62 (m, 1 H)	C-24	28.83
H-25	1.62 (m, 1 H)	C-25	31.40
H-26	3.38 (m, 1 H), 3.47 (m, 1 H)	C-26	66.84
H-27	0.79 (s, CH_3)	C-27	17.16

Table S10: ^{1}H and ^{13}C NMR data for glycoside **12** ($CDCl_3$).

Proton	δ (ppm) and *J* (Hz)	Carbon	δ (ppm)
H-1’	4.65 (d, 1 H, $J_{1,2}$ 8.2)	C-1’	100.33
H-2’	2.94 (t, 1 H, $J_{1,2}$ 9.0, $J_{2,3}$ 9.7)	C-2’	60.84
H-3’	3.53 (m, 1 H)	C-3’	68.57
H-4’	4.06 (m, 1 H)	C-4’	68.04
H-5’	3.50 (m, 1 H)	C-5’	74.40
H-6’	3.80 (m, 1 H), 3.96 (m, 1 H, J_{gem} 8.2)	C-6’	62.76
$–(C\mathbf{H}_2CH_3)_2$	2.78 (m, 4 H)	$–(\mathbf{C}H_2CH_3)_2$	44.71
$–(CH_2C\mathbf{H}_3)_2$	1.08 (t, 6 H, *J* 7.0)	$–(CH_2\mathbf{C}H_3)_2$	15.11
H-1	1.14 (m, 1 H), 1.83 (m, 1 H)	C-1	37.15
H-2	1.60 (m, 1 H), 1.67 (m, 1 H)	C-2	31.38
H-3	3.52 (m, 1 H)	C-3	78.70
H-4	2.31 (m, 2 H)	C-4	39.22
		C-5	140.56
H-6	5.35 (m, 1 H, *J* 4.7)	C-6	121.72
H-7	1.52 (m, 1 H), 2.01 (m, 1 H)	C-7	32.08
H-8	1.62 (m, 1 H)	C-8	30.30
H-9	0.94 (m, 1 H)	C-9	50.08
		C-10	36.90
H-11	1.47 (m, 2 H)	C-11	20.82
H-12	1.19 (m, 1 H), 1.74 (m, 1 H)	C-12	39.75
		C-13	40.27
H-14	1.12 (m, 1 H)	C-14	56.50
H-15	1.30 (m, 1 H), 1.99 (m, 1 H)	C-15	31.85
H-16	4.42 (q, 1 H, *J* 7.5)	C-16	80.82
H-17	1.79 (m, 1 H)	C-17	62.08
H-18	0.78 (s, CH_3)	C-18	16.29
H-19	1.03 (s, CH_3)	C-19	19.41
H-20	1.86 (m, 1 H)	C-20	41.61
H-21	0.98 (d, CH_3, *J* 7.0)	C-21	14.52
		C-22	109.30
H-23	1.96 (m, 2 H)	C-23	30.03
H-24	1.45 (m, 1 H), 1.60 (m, 1 H)	C-24	28.80
H-25	1.63 (m, 1 H)	C-25	31.40
H-26	3.38 (m, 1 H), 3.47 (m, 1 H)	C-26	66.86
H-27	0.80 (s, CH_3)	C-27	17.13

Table S11: ^{1}H and ^{13}C NMR data for glycoside **13** ($CDCl_3$:CD_3OD = 1:1).

Proton	δ (ppm) and *J* (Hz)	Carbon	δ (ppm)
H-1'	4.68 (d, 1 H, $J_{1,2}$ 8.4)	C-1'	100.26
H-2'	2.87 (t, 1 H, $J_{1,2}$ 8.7, $J_{2,3}$ 10.2)	C-2'	61.33
H-3'	3.50 (dd, 1 H, $J_{3,4}$ 2.6, $J_{2,3}$ 10.6)	C-3'	69.02
H-4'	3.99 (m, 1 H)	C-4'	67.25
H-5'	3.46 (m, 1 H)	C-5'	74.77
H-6'	3.72 (m, 2 H)	C-6'	61.18
–(C**H**$_2$CH$_2$CH$_3$)$_2$	2.61 (m, 2 H), 2.73 (m, 2 H)	–(**C**H$_2$CH$_2$CH$_3$)$_2$	53.11
–(CH$_2$C**H**$_2$CH$_3$)$_2$	1.41 (dd, 2 H, *J* 7.5, J_{gem} 12.0), 1.53 (d, 2 H)	–(CH$_2$**C**H$_2$CH$_3$)$_2$	22.25
–(CH$_2$CH$_2$C**H**$_3$)$_2$	0.88 (t, 6 H, *J* 7.0)	–(CH$_2$CH$_2$**C**H$_3$)$_2$	11.14
H-1	1.08 (m, 1 H), 1.83 (m, 1 H)	C-1	37.08
H-2	1.58 (m, 1 H), 1.90 (m, 1 H)	C-2	29.74
H-3	3.56 (m, 1 H)	C-3	78.44
H-4	2.25 (m, 1 H), 2.36 (m, 1 H, J_{gem} 12.0	C-4	39.18
		C-5	140.42
H-6	5.36 (m, 1 H)	C-6	121.49
H-7	1.49 (m, 1 H), 2.00 (m, 1 H)	C-7	31.90
H-8	1.63 (m, 1 H)	C-8	30.05
H-9	0.97 (m, 1 H)	C-9	50.13
		C-10	36.72
H-11	1.53 (m, 2 H)	C-11	20.67
H-12	1.19 (dd, 1 H, *J* 4.2, J_{gem} 12.6), 1.24 (m, 1 H)	C-12	39.60
		C-13	40.14
H-14	1.13 (m, 1 H)	C-14	56.43
H-15	1.30 (dd, 1 H, *J* 4.9, J_{gem} 10.1), 1.99 (dd, 1 H, *J* 6.1, J_{gem} 13.2)	C-15	31.48
H-16	4.39 (q, 1 H, *J* 7.4)	C-16	80.92
H-17	1.75 (m, 1 H)	C-17	62.06
H-18	0.79 (s, CH$_3$)	C-18	16.52
H-19	1.02 (s, CH$_3$)	C-19	18.94
H-20	1.83 (m, 1 H)	C-20	41.54
H-21	0.95 (d, CH$_3$, *J* 6.9)	C-21	13.89
		C-22	109.46
H-23	1.60 (m, 1 H), 1.69 (m, 1 H)	C-23	31.11
H-24	1.43 (m, 1 H), 1.63 (m, 1 H)	C-24	28.51
H-25	1.61 (m, 1 H)	C-25	31.37
H-26	3.34 (m, 1 H), 3.45 (m, 1 H)	C-26	66.67
H-27	0.78 (s, CH$_3$, *J* 7.1)	C-27	15.83

References

1. Clinical and Laboratory Standards Institute (CLSI), *Methods for Dilution Antimicrobial Susceptibility Tests for Bacteria That Grow Aerobically; Approved Standard-Ninth Edition.*, vol. 32, no. 2. 2012.
2. Clinical and Laboratory Standards Institute (CLSI), *Reference method for broth dilution antifungal susceptibility testing of yeasts; Approved Standards-Second Edition, in CLSI document M27-2A 2002, CLSI Pennsylvania, USA*, vol. 22, no. 15. 2002.
